# Antihypertensive treatments in adult autosomal dominant polycystic kidney disease: network meta-analysis of the randomized controlled trials

**DOI:** 10.18632/oncotarget.6452

**Published:** 2015-12-02

**Authors:** Cheng Xue, Chenchen Zhou, Bing Dai, Shengqiang Yu, Chenggang Xu, Zhiguo Mao, Chaoyang Ye, Dongping Chen, Xuezhi Zhao, Jun Wu, Wansheng Chen, Changlin Mei

**Affiliations:** ^1^ Department of Nephrology, Shanghai Changzheng Hospital, Second Military Medical University, Shanghai, China; ^2^ Department of Nephrology, PLA 309 Hospital, Beijing, China; ^3^ Department of Pharmacy, Shanghai Changzheng Hospital, Second Military Medical University, Shanghai, China

**Keywords:** polycystic kidney disease, autosomal dominant, antihypertensive drug, glomerular filtration rate, urinary albumin excretion, Pathology Section

## Abstract

**Background:**

Blood pressure (BP) control is one of the most important treatments of Autosomal dominant polycystic kidney disease (ADPKD). The comparative efficacy of antihypertensive treatments in ADPKD patients is inconclusive.

**Methods:**

Network meta-analysis was used to evaluate randomized controlled trials (RCT) which investigated antihypertensive treatments in ADPKD. PubMed, Embase, Ovid, and Cochrane Collaboration were searched. The primary outcome was estimated glomerular filtration rate (eGFR). Secondary outcomes were serum creatinine (Scr), urinary albumin excretion (UAE), systolic BP (SBP), diastolic BP (DBP), mean artery pressure (MAP) and left ventricular mass index (LVMI).

**Results:**

We included 10 RCTs with 1386 patients and six interventions: angiotensin-converting enzyme inhibitors (ACEI), Angiotensin II receptor blocker (ARB), combination of ACEI and ARB, calcium channel blockers (CCB), β-blockers and dilazep. There was no difference of eGFR in all the treatments in both network and direct comparisons. No significant differences of Scr, SBP, DBP, MAP, and LVMI were found in network comparisons. However, ACEI signiﬁcantly reduced SBP, DBP, MAP and LVMI when compared to CCB. Significantly increased UAE was observed in CCB compared with ACEI or ARB. Bayesian probability analysis found ARB ranked first in the surrogate measures of eGFR, UAE and SBP.

**Conclusions:**

There is little evidence to detect differences of antihypertensive treatments on kidney disease progression in ADPKD patients. More RCTs will be needed in the future. Use of ARB may be an optimal choice in clinical practice.

## INTRODUCTION

Autosomal dominant polycystic kidney disease (ADPKD) is characterized by continuous enlargement of kidney cysts. ADPKD is the most common hereditary nephropathy with prevalence from 1/1000 to 1/400 [1]. ADPKD patients develop hypertension early, which increases the renal progression. ADPKD patients with hypertension have faster and greater annual rates of total kidney volume (TKV) growth, and an increased prevalence of cardiovascular complications when compared with the normotensive patients. Healthcare for ADPKD mainly focuses on hypertension to reduce mortality and morbidity. Currently, blood pressure (BP) control is one of the most important clinical treatments of ADPKD.

The renin–angiotensin–aldosterone system (RAAS) plays an important role in hypertension pathogenesis in ADPKD [2]. RAAS inhibitors (RASI) include Angiotensin converting enzyme inhibitor (ACEI) and Angiotensin II receptor blocker (ARB). RASIs have been proved to slow renal progression in non-diabetes chronic kidney disease (CKD), and are widely used in clinical practice of ADPKD. Besides, calcium channel blockers (CCBs), β-blockers, dilazep and the diuretics also were used in ADPKD with hypertension [3–5]. There was no difference in renal function between ACEI and placebo [6]. Kanno et al. [7] found CCB showed higher creatinine clearance compared with ACEI. However, a randomized controlled trial (RCT) found renal function was similar between amlodipine and enalapril [8]. Recently, the Halt Progression of Polycystic Kidney Disease studies (HALT-PKD) [1,2] observed a negative effect of the combination of ACEI and ARB on renal function compared with ACEI monotherapy.

Each RCT just contained only two or three drugs. It is hard to get a head-to-head outcome comparing the drugs of interest or get all the drugs to integrate some specific effects together [9]. This study aimed to use network and traditional meta-analysis to assess the direct and indirect effects of antihypertensive treatments in ADPKD.

## RESULTS

**Figure 1:**
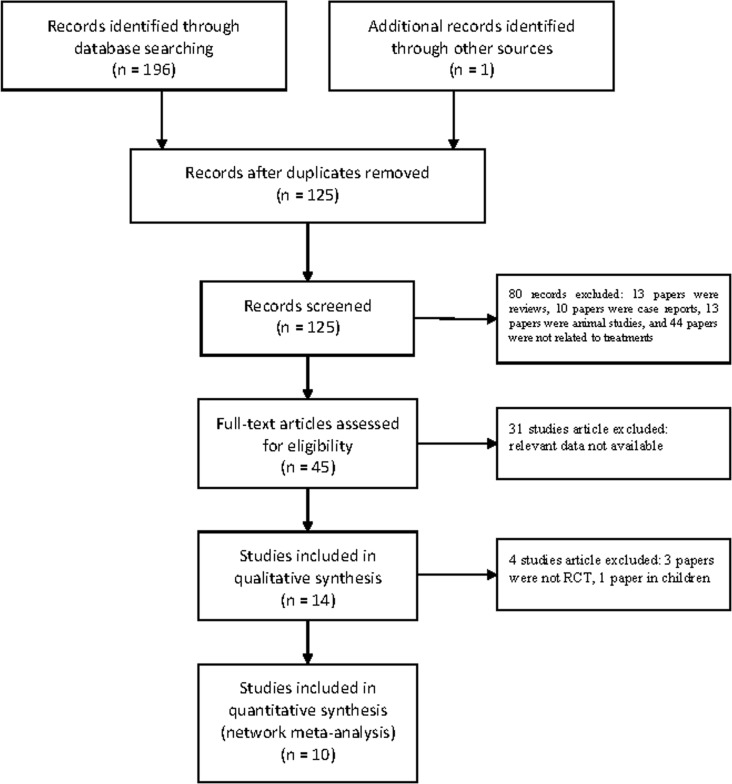
Flow chart of the included studies

**Figure 2:**
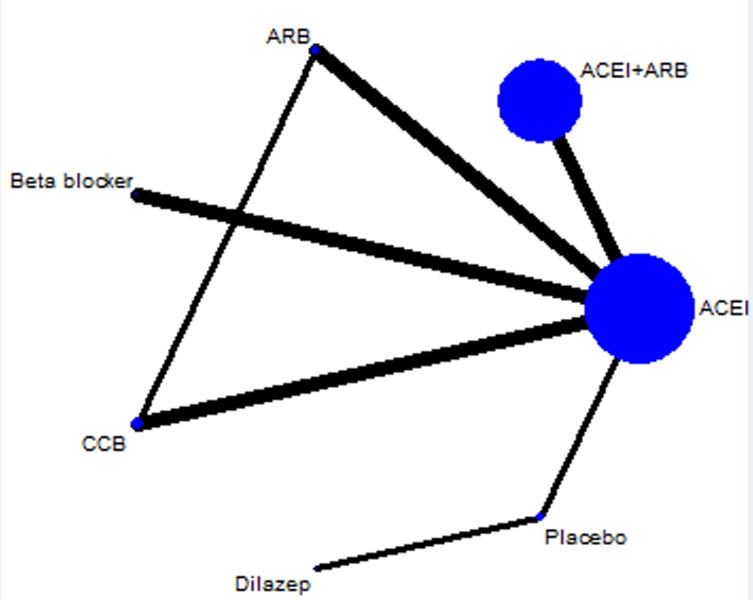
Network of antihypertensive drugs in ADPKD The size of treatment nodes (blue circles) reﬂected the number of studies. The thickness of lines represented the number of trials in that comparison. ARB: angiotensin-receptor blocker. ACEI: angiotensin-converting-enzyme inhibitor. CCB: calcium-channel blocker.

Ten RCTs with 1,386 participants were included after assessment of 45 full-text articles and 197 records [1–8,10–15]. Electronic searching process was shown in the ﬂowchart (Figure 1). Eight trials were two-grouped, and two trials were four-grouped. The network of included treatment comparisons was shown in Figure 2. ACEIs were the most frequently studied agents. The baseline characteristics were summarized in Table 1. The mean follow-up time was about four years (range 0.5–8 years). Male/female proportion was balanced in all trials. The hypertension criteria in the studies was > 140/90 mm Hg. Two studies [6,15] divided patients into hypertension and normal BP groups.

**Table 1:** Characteristics of the included trials

Studies	Country	Randomization	Duration	Groups	Number	Male/female	Age	GFR	SBP	DBP	LVMI	Scr	UAE	Outcomes
		Setting	(months)			(N)	(years)	ml/min	mmHg	mmHg	(g/m^2^)	(mg/dl)	(mg/24h)	
Ecder	USA	RCT	60	Amlodipine	12	8/4	43±3	83±5	140±5	93±3	NA	1.18±0.06	68±21	①②③④⑤⑥
2000		Single center		Enalapril	12	5/7	41±2	77±6	136±3	94±3	NA	1.19±0.09	23±4	
Schrier	USA	RCT	84	Enalapril	49	NA	43±9	79±31	NA	NA	159±25	NA	NA	①⑦
2002		Single center		Amlodipine	20	NA	41±7	84±24	NA	NA	159±25	NA	NA	
van Dijk	Netherlands	DB RCT	36	Enalapril	13	5/8	40±3	80±9	144±3	98±2	NA	1.35±0.14	39±31	①③⑥
2003		Single center		Atenolol	15	5/10	33±3	92±9	144±3	96±1	NA	1.27±0.14	33±28	
				Enalapril	32	11/21	36±2	103±2	133±2	88±2	NA	1.00±0.02	46±68	
				Placebo	29	14/15	37±2	103±1	133±2	88±1	NA	1.00±0.01	39±50	
Nutahara	Japan	RCT	36	Amlodipine	25	13/12	48.4±5	71.9±20.5	NA	NA	NA	1.22±0.34	148±187	①②③④⑤
2005		Multicenter		Candesartan	24	13/11	47.3±5	69.8±24.6	NA	NA	NA	1.12±0.30	116±102	
Nakamura	Japan	RCT	6	Dilazep	6	NA	NA	106.4±12.2	112±16	NA	NA	0.80±0.30	130±52	①②③④⑤
2005		Single center		Placebo	6	NA	NA	102.8±13.8	114±14	NA	NA	0.90±0.40	132±56	
				Dilazep	5	NA	NA	102.8±16.0	158±12	NA	NA	0.90±0.40	142±48	
				Placebo	5	NA	NA	96.2±12.8	156±14	NA	NA	1.00±0.20	136±42	
Zeltner	Germany	RCT	36	Ramipril	17	10/7	40.7±2.2	88.0±9.5	143±2	93±2	97.6±6.1	1.30±0.19	64.0±21.6	①②③④⑤⑥⑦
2008		Single center		Metoprolol	20	7/13	40.0±2.2	87.3±6.4	142±2	90±2	95.0±4.2	1.16±0.09	75.3±22.8	
Ulusoy	Turkey	RCT	12	Losartan	19	6/13	51.4±10.3	75.9±29.8	156.3±15.7	98.7±12.5	117.3±18.8	1.25±0.57	NA	①②④⑤⑥⑦
2010		Single center		Ramipril.	13	7/6	47.7±7.4	80.1± 9.3	150±19.6	94.2±4.9	120.7±16.3	1.40±0.77	NA	
Nakamura	Japan	RCT	12	Telmisartan	10	6/4	56.6±6.4	65.9± 6.4	158± 6	96± 5	NA	0.80±0.09	90.2±32.5	②③④⑤
2012		Single center		Enalapril	10	5/5	58.1±5.6	67.9±4.5	159±6	97± 6	NA	0.76±0.11	92.2±31.0	
Schrier	USA	DB RCT	96	Lisinopril+telmisartan	273	141/142	37.0±8.3	90.4±17.5	NA	NA	64.1±13.2	NA	19.3±10.2	①③④⑤⑦
2014		Multicenter		Lisinopril+placebo	285	142/143	36.3±8.3	92.6±17.4	NA	NA	63.7±12.9	NA	17.6±10.3	
Torres	USA	DB RCT	96	Lisinopril+telmisartan	244	115/129	48.6±8.5	48.5±11.5	NA	NA	NA	1.5±0.4	29.7±29.2	①③④⑤⑥
2014		Multicenter		Lisinopril+placebo	242	120/122	48.9±8.1	47.9±12.2	NA	NA	NA	1.6±0.4	28.1±30.6	

SB, single-blinded; DB, double-blinded; RCT, randomized controlled trial; eGFR glomerular filtration rate; SBP, systolic blood pressure; DBP, diastolic blood pressure; LVMI, left ventricular mass index; UAE, urinary albumin excretion; Scr, serum creatinine; NA, not available; Outcomes: ① eGFR, ② Scr, ③ UAE, ④ SBP, ⑤ DBP, ⑥ MAP, ⑦ LVMI.

**Figure 3:**
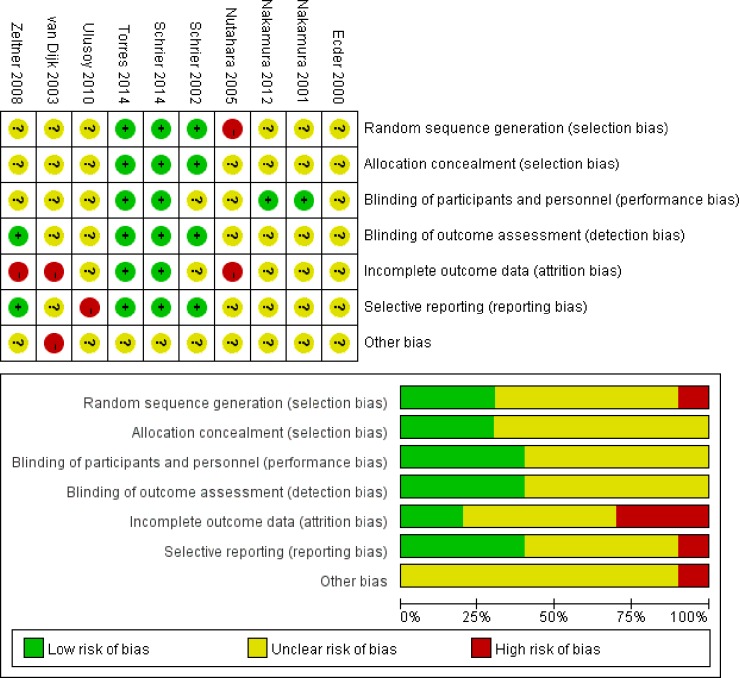
Risk of bias summary for the included studies

The overall risk of bias of the included studies was shown in Figure 3. Random sequence generation was adequate in two studies. 60% studies did not present adequate blinding. Only three studies used intention-to-treat analyses. Predeﬁned endpoints were reported fully in four studies.

Network comparisons for primary outcome eGFR were shown in Table 2. There was no difference of eGFR in all the treatments (seven studies, five treatments, Supplemental Figure 1). There was no increased eGFR with ACEI, ARB, or ACEI+ARB when compared with β-blocker or CCB either in the consistency model or in the inconsistency model.

**Table 2:** The effects of the antihypertensive treatments in the eGFR

ACEI	−0.81 (−16.34, 14.34)	3.79 (−19.07, 26.53)	1.61 (−12.34, 22.27)	7.75 (−14.21, 28.48)
0.95 (−14.77, 17.07)	ACEI+ARB	4.69 (−21.82, 32.15)	2.35 (−16.75, 29.26)	3.65 (−24.84, 32.68)
−5.88 (−26.54, 18.72)	−6.66 (−32.71, 22.42)	ARB	−1.71 (−28.01, 28.71)	−1.15 (−22.28, 20.72)
−1.95 (−23.22, 12.13)	−2.75 (−30.53, 17.21)	3.59 (−29.61, 28.07)	β-blocker	0.61 (−30.58, 28.61)
−6.23 (−26.17, 15.48)	−7.19 (−32.86, 19.76)	−0.22 (−22.72, 20.78)	−4.08 (−27.62, 26.48)	CCB

Data was listed as MD with 95% CI. Effect estimates from the network meta-analysis in the consistency model occupy the bottom left part of the diagram, and the estimates from the inconsistency model occupy the top right part of the diagram. The diagonal corresponds to the comparison. The data should be read from left to right in the bottom left part of the diagram, and from right to left in the top right part of the diagram.

Table 3 showed network comparisons for the Scr. No significant difference was found in all the treatments (five studies, four treatments, Supplemental Figure 2). There was no decreased Scr with ACEI or ARB when compared with β-blocker or CCB either in the consistency model or in the inconsistency model. Table 4 showed the network comparisons for the UAE (seven studies, five treatments, Supplemental Figure 3). UAE in ACEI, ARB, ACEI+ARB and β-blocker did not differ, but UAE tended to be higher in CCB. There was increased UAE with CCB when compared with all the RASI treatments and β-blocker in the consistency model. However, we did not find increased UAE in CCB than β-blocker in the inconsistency model (MD 169.66, 95% CI −11.59, 351.46). Table 5 showed the network comparisons for the SBP (seven studies, five treatments, Supplemental Figure 4). No significant difference was observed in all the treatments either in the consistency model or in the inconsistency model. Table 6 showed network comparisons for the DBP (seven studies, five treatments, Supplemental Figure 5). DBP in all the treatments did not differ. Table 7 showed the network comparisons for the MAP (five studies, five treatments, Supplemental Figure 6). No significant difference was observed in all the treatments either in the consistency model or in the inconsistency model. Table 8 showed the network comparisons for the LVMI (four studies, five treatments, Supplemental Figure 7). LVMI lowering effect was similar in all the treatments either in the consistency model or in the inconsistency model.

**Table 3:** The effects of the antihypertensive treatments in the Scr

ACEI	−0.15 (−0.58, 0.29)	−0.18 (−0.75, 0.38)	0.02 (−0.36, 0.53)
0.16 (−0.25, 0.57)	ARB	−0.03 (−0.74, 0.67)	0.26 (−0.27, 0.83)
0.17 (−0.40, 0.77)	0.00 (−0.69, 0.71)	β-blocker	0.29 (−0.46, 1.09)
−0.04 (−0.53, 0.35)	−0.21 (−0.72, 0.25)	−0.21 (−1.00, 0.46)	CCB

Data was listed as MD with 95% CI. Effect estimates from the network meta-analysis in the consistency model occupy the bottom left part of the diagram, and the estimates from the inconsistency model occupy the top right part of the diagram. The diagonal corresponds to the comparison. The MD and 95% CI for the comparisons should be read from left to right. The data should be read from left to right in the bottom left part of the diagram, and from right to left in the top right part of the diagram.

**Table 4:** The effects of the antihypertensive treatments in the UAE

ACEI	−4.78 (−98.28, 86.41)	−26.38 (−158.54, 75.14)	9.62 (−84.67, 102.61)	**142.47 (34.43, 266.82)**
4.87 (−75.73, 97.85)	ACEI+ARB	−21.98 (−188.63, 117.60)	14.48 (−117.15, 143.84)	**184.71 (3.29, 366.13)**
29.95 (−61.87, 145.72)	25.54 (−104.17, 164.39)	ARB	35.57 (−98.63, 199.18)	**209.14 (68.09, 367.05)**
−9.49 (−96.03, 75.27)	−14.76 (−140.97, 103.48)	−39.27 (−185.32, 78.06)	β-blocker	169.66 (−11.59, 351.46)
**−146.03 (−263.33, −47.16)**	**−150.86 (−305.70, −26.34)**	**−177.55 (−317.05, −74.83)**	**−135.99 (−284.14, −3.62)**	CCB

Data was listed as MD with 95% CI. Effect estimates from the network meta-analysis in the consistency model occupy the bottom left part of the diagram, and the estimates from the inconsistency model occupy the top right part of the diagram. The diagonal corresponds to the comparison. The MD and 95% CI for the comparisons should be read from left to right. Significant results are underlined and in bold. The data should be read from left to right in the bottom left part of the diagram, and from right to left in the top right part of the diagram.

**Table 5:** The effects of the antihypertensive treatments in the SBP

ACEI	−0.63 (−4.68, 4.14)	−1.60 (−6.37, 3.05)	1.00 (−4.36, 6.33)	4.70 (−1.19, 10.13)
0.66 (−4.21, 4.75)	ACEI+ARB	−1.01 (−7.65, 5.05)	1.67 (−5.68, 8.34)	5.07 (−3.93, 12.81)
1.50 (−3.18, 6.22)	0.85 (−5.22, 7.56)	ARB	2.56 (−4.44, 9.66)	6.08 (−2.23, 13.75)
−1.00 (−6.23, 4.32)	−1.67 (−8.19, 5.68)	−2.48 (−9.35, 4.55)	β-blocker	3.46 (−5.77, 11.95)
−4.70 (−9.96, 1.30)	−5.40 (−11.90, 2.62)	−6.17 (−12.87, 1.00)	−3.74 (−11.02, 4.35)	CCB

Data was listed as MD with 95% CI. Effect estimates from the network meta-analysis in the consistency model occupy the bottom left part of the diagram, and the estimates from the inconsistency model occupy the top right part of the diagram. The diagonal corresponds to the comparison. The MD and 95% CI for the comparisons should be read from left to right. The data should be read from left to right in the bottom left part of the diagram, and from right to left in the top right part of the diagram.

**Table 6:** The effects of the antihypertensive treatments in the DBP

ACEI	−1.31 (−8.62, 3.40)	−0.64 (−5.95, 4.99)	−1.00 (−8.38, 6.35)	2.75 (−4.96, 9.68)
1.13 (−3.31, 8.38)	ACEI+ARB	0.71 (−6.04, 10.25)	0.21 (−8.00, 11.17)	3.80 (−7.42, 15.63)
0.69 (−4.95, 5.80)	−0.53 (−10.22, 5.86)	ARB	−0.35 (−9.79, 8.72)	2.94 (−8.11, 12.93)
0.93 (−6.36, 8.31)	0.04 (−11.16, 7.80)	0.26 (−8.60, 9.57)	β-blocker	3.34 (−9.22, 14.97)
−2.81 (−9.45, 4.74)	−3.84 (−14.08, 4.08)	−3.44 (−11.26, 5.65)	−3.77 (−13.41, 6.82)	CCB

Data was listed as MD with 95% CI. Effect estimates from the network meta-analysis in the consistency model occupy the bottom left part of the diagram, and the estimates from the inconsistency model occupy the top right part of the diagram. The diagonal corresponds to the comparison. The MD and 95% CI for the comparisons should be read from left to right. The data should be read from left to right in the bottom left part of the diagram, and from right to left in the top right part of the diagram.

**Table 7:** The effects of the antihypertensive treatments in the MAP

ACEI	−4.85 (−17.71, 8.31)	−2.55 (−10.30, 5.13)	0.83 (−3.68, 5.83)	3.04 (−3.97, 9.98)
4.26 (−8.85, 17.31)	ACEI+ARB	2.18 (−12.90, 17.24)	5.65 (−8.14, 19.72)	7.85 (−6.72, 22.43)
2.70 (−5.13, 10.37)	−1.59 (−16.66, 13.60)	ARB	3.39 (−5.54, 12.63)	5.62 (−4.93, 16.08)
−0.83 (−5.78, 3.70)	−5.13 (−19.00, 8.59)	−3.55 (−12.69, 5.48)	β-blocker	2.20 (−6.06, 10.42)
−2.93 (−9.99, 4.12)	−7.27 (−22.16, 7.37)	−5.65 (−15.88, 4.73)	−2.14 (−10.48, 6.72)	CCB

Data was listed as MD with 95% CI. Effect estimates from the network meta-analysis in the consistency model occupy the bottom left part of the diagram, and the estimates from the inconsistency model occupy the top right part of the diagram. The diagonal corresponds to the comparison. The MD and 95% CI for the comparisons should be read from left to right. The data should be read from left to right in the bottom left part of the diagram, and from right to left in the top right part of the diagram.

**Table 8:** The effects of the antihypertensive treatments in the LVMI

ACEI	0.41 (−34.15, 35.78)	5.14 (−31.10, 40.07)	−2.58 (−37.76, 31.34)	27.10 (−9.23, 64.91)
−0.29 (−34.40, 33.07)	ACEI+ARB	4.82 (−45.86, 53.24)	−2.79 (−52.75, 44.60)	26.73 (−22.61, 77.88)
−5.14 (−41.44, 31.46)	−4.85 (−54.72, 44.27)	ARB	−7.73 (−56.80, 41.13)	21.73 (−27.05, 74.00)
2.13 (−32.01, 35.90)	2.48 (−45.68, 51.30)	7.27 (−42.40, 56.31)	β-blocker	29.70 (−19.46, 82.01)
−27.08 (−63.78, 8.59)	−26.60 (−76.63, 22.67)	−21.36 (−74.09, 28.23)	−29.11 (−79.47, 19.34)	CCB

Data was listed as MD with 95% CI. Effect estimates from the network meta-analysis in the consistency model occupy the bottom left part of the diagram, and the estimates from the inconsistency model occupy the top right part of the diagram. The diagonal corresponds to the comparison. The MD and 95% CI for the comparisons should be read from left to right. The data should be read from left to right in the bottom left part of the diagram, and from right to left in the top right part of the diagram.

**Figure 4:**
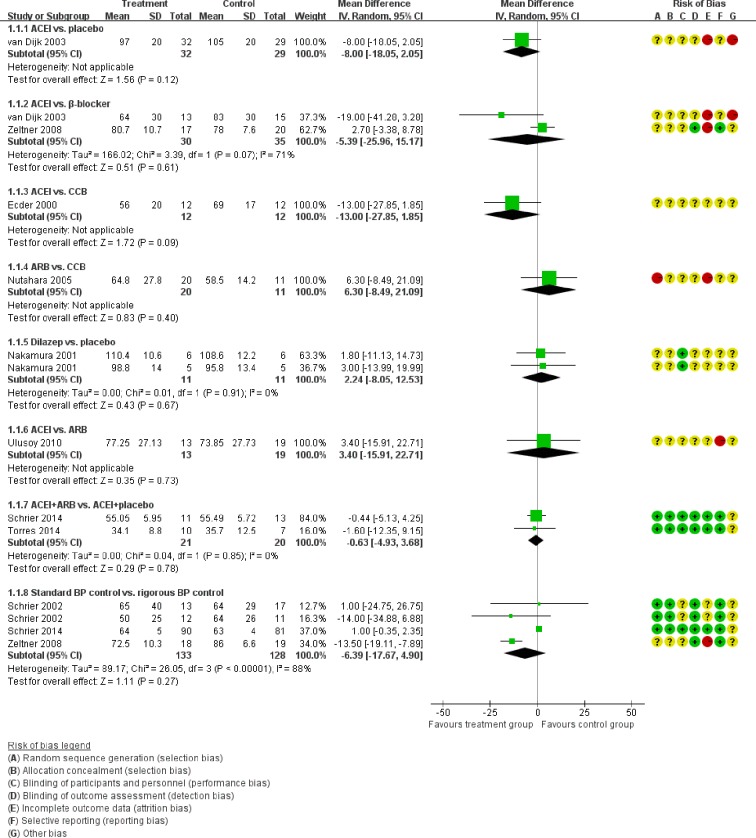
Meta-analysis of all the antihypertensive treatments in eGFR

In direct comparisons of the primary outcome, the results were almost similar to the network comparisons. There were no statistical difference in the eGFR across the following comparisons (Figure 4): ACEI vs. placebo (one study, 61 participants, MD −8.00, 95% CI −18.05, 2.05, *P*=0.12); ACEI vs. β-blocker (two studies, 65 participants, MD −5.39, 95% CI −25.96, 15.17, *P*=0.61), ACEI vs. CCB (one study, 24 participants, MD −13.00, 95% CI −27.85, 1.85, *P*=0.09), ARB vs. CCB (one study, 31 participants, MD 6.30, 95% CI −8.49, 21.09, *P*=0.40), ACEI vs. ARB (one study, 32 participants, MD 3.40, 95% CI −15.91, 22.71, *P*=0.78), Dilazep vs. placebo (one study, 22 participants, MD 2.24, 95% CI −8.05, 12.53, *P*=0.67), and ACEI+ARB vs. ACEI (two studies, 41 participants, MD −0.63, 95% CI −4.93, 3.68, *P*=0.61).

**Figure 5:**
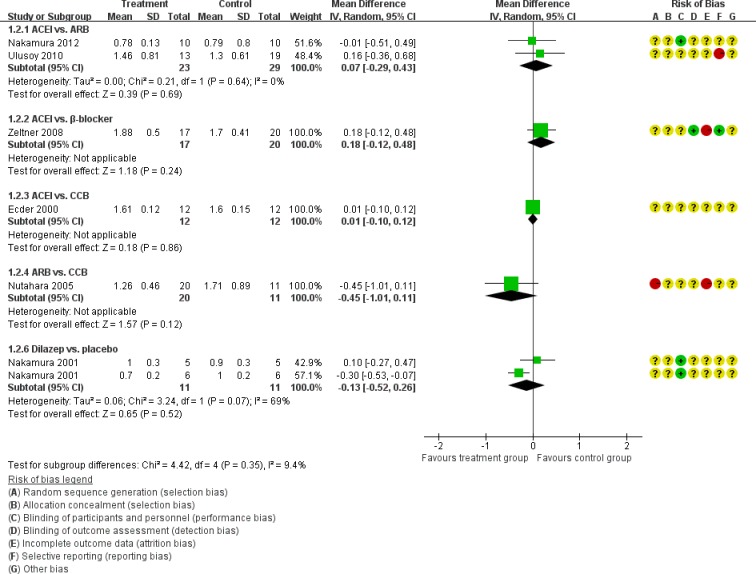
Meta-analysis of all the antihypertensive treatments in Scr

**Figure 6:**
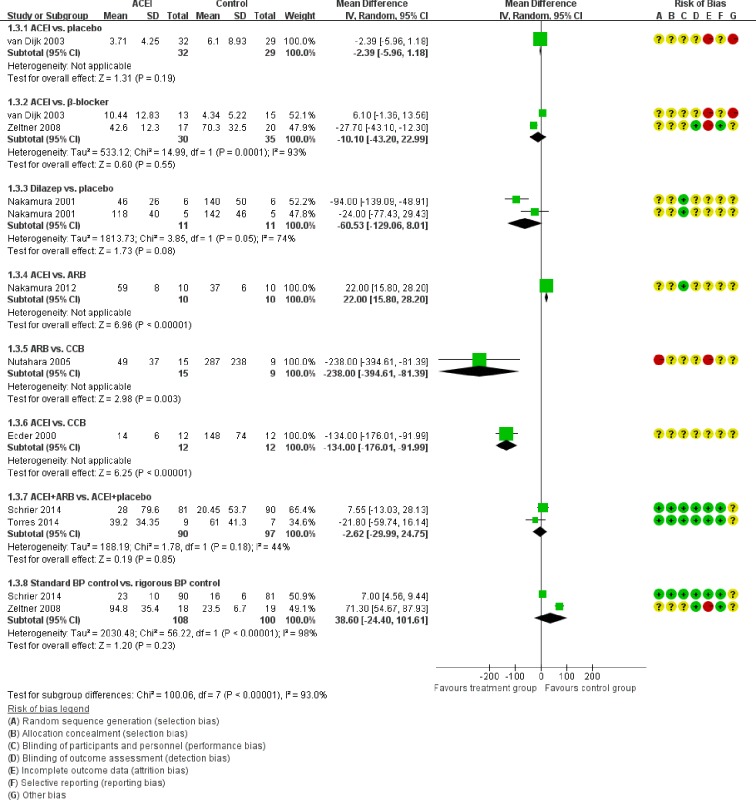
Meta-analysis of all the antihypertensive treatments in UAE

**Figure 7:**
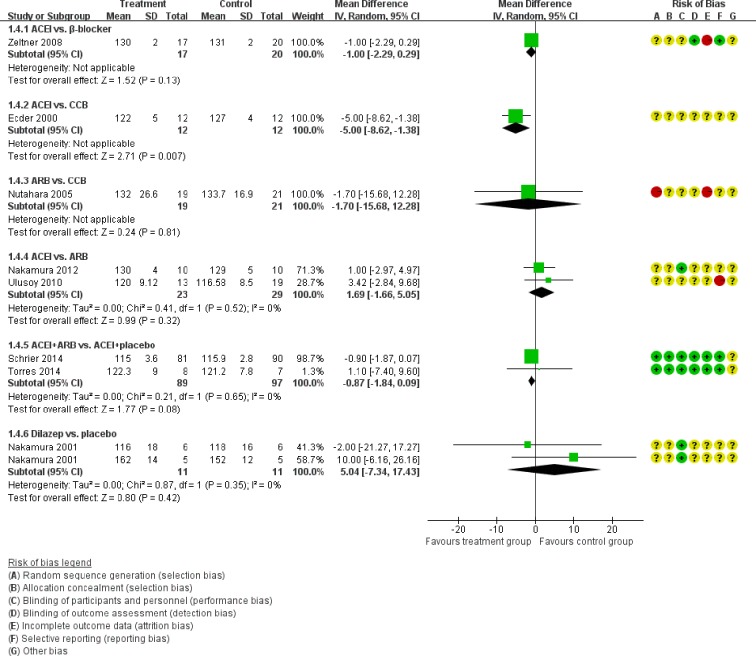
Meta-analysis of all the antihypertensive treatments in SBP

**Figure 8:**
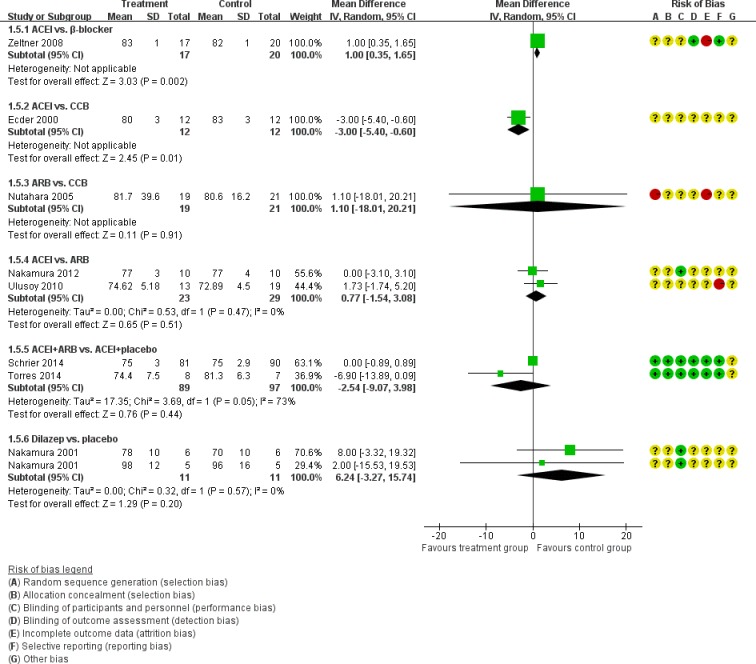
Meta-analysis of all the antihypertensive treatments in DBP

**Figure 9:**
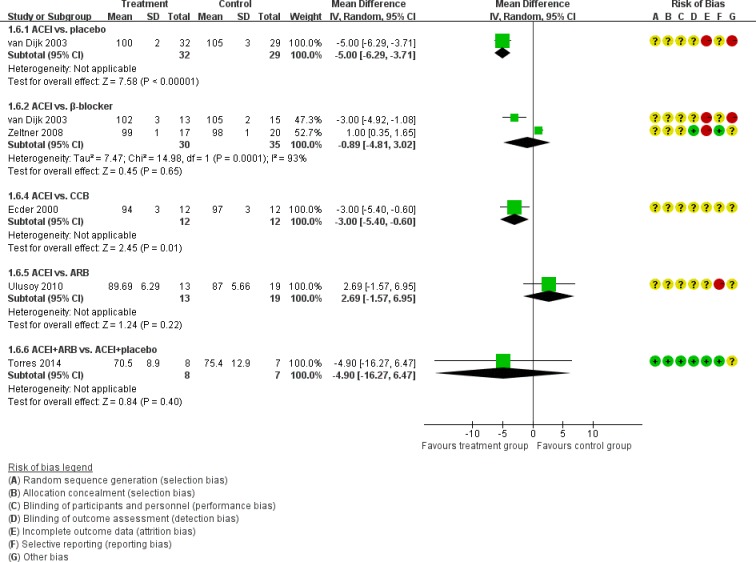
Meta-analysis of all the antihypertensive treatments in MAP

**Figure 10:**
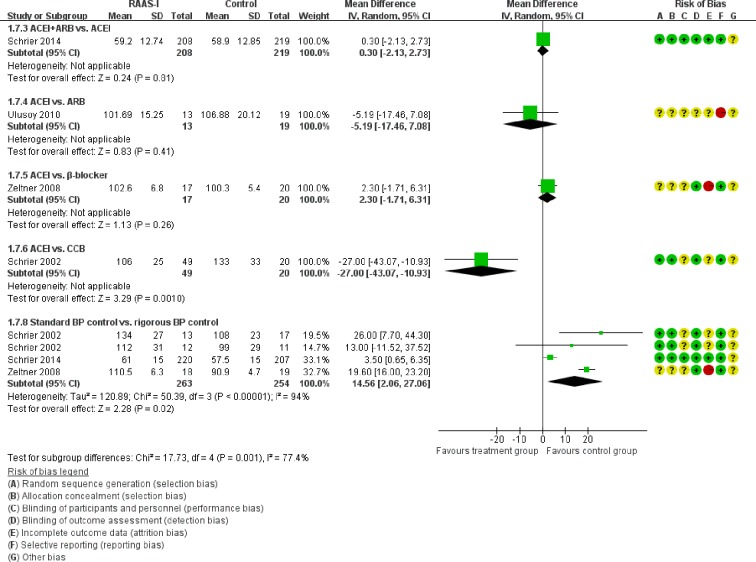
Meta-analysis of all the antihypertensive treatments in LVMI

Figure 5 showed the direct comparisons of Scr. No significant difference was observed in direct comparisons of Scr. Figure 6 showed the direct comparisons of UAE. Nutahara et al. [3] reported ARB signiﬁcantly decreased UAE (24 participants, MD −238.00, 95% CI −394.61, −81.39, *P*=0.003) compared with CCB. Ecder et al. [8] showed that the ACEI decreased UAE significantly compared to the CCB (24 participants, MD −134.00, 95% CI −176.01, −91.99, *P*<0.00001). Furthermore, the ARB was associated with lower UAE compared with the ACEI (one study, 20 participants, MD −22, 95% CI −28.20, −15.80, *P*<0.00001). Figure 7 showed the direct comparisons of SBP. SBP is lower after the treatment of ACEI than the CCB (one study, 24 participants, MD −5.00, 95% CI −8.62, −1.38, *P*=0.007). Figure 8 showed the direct comparisons of DBP. DBP is also lower after the treatment of ACEI than the CCB (one study, 24 participants, MD −3.00, 95% CI −5.40, −0.60, *P*=0.01). However, the ACEI significantly increased the DBP compared to the β-blocker (one study, 37 participants, MD 1.00, 95% CI 0.35, 1.65, *P*=0.002). Figure 9 showed the direct comparisons of MAP. MAP is lower in the treatment of ACEI compared with the CCB (one study, 24 participants, MD −3.00, 95% CI −5.40, −0.60, *P*=0.007) and the placebo (one study, 61 participants, MD −5.00, 95% CI −6.29, −3.71, *P*<0.00001). Figure 10 showed the direct comparisons of LVMI. LVMI was lower after the treatment of ACEI compared with the CCB (one study, 69 participants, MD −27.00, 95% CI −43.07, −10.93, *P*=0.001).

Then we performed direct comparisons between the rigorous BP control group (target < 120/80 mmHg) and the standard BP control group (target 120/80-140/90 mm Hg). The results found the rigorous BP group was associated with a greater decrease in LVMI (three studies, 517 participants, MD −14.56, 95% CI −27.06, −2.06, *P*=0.02) compared with the standard BP group (Figure 10). However, the eGFR was similar between the two groups (three studies, 261 participants, MD −6.39, 95% CI −17.67, 4.90, *P*=0.27) (Figure 4). UAE tended to be less in the rigorous BP group (two studies, 208 participants, MD −38.6, 95% CI −101.61, 24.4, *P*=0.23), but the result was not significant (Figure 6).

Bayesian probability analysis found the ARB had 34% probability to be the best treatment in eGFR. The ranking sequence was shown in Table 9. ARB also ranked first in the UAE and the SBP. B-blocker ranked first in the Scr and the LVMI. ACEI+ARB ranked first in the DBP and the MAP.

**Table 9:** The rank sequence of the antihypertensive treatments in the outcomes

Drug	eGFR	Scr	UAE	SBP	DBP	MAP	LVMI
ACEI	Rank 3	Rank 3	Rank 3	Rank 3	Rank 3	Rank 3	Rank 2
ACEI+ARB	Rank 4		Rank 2	Rank 2	**Rank 1**	**Rank 1**	Rank 3
ARB	**Rank 1**	Rank 2	Rank 1	**Rank 1**	Rank 4	Rank 2	Rank 4
β-blocker	Rank 5	**Rank 1**	Rank 4	Rank 4	Rank 2	Rank 4	**Rank 1**
CCB	Rank 2	Rank 4	Rank 5	Rank 5	Rank 5	Rank 5	Rank 5

Rank 1 was the best. The bigger number of the rank, the worse rank. Rank 1 was underlined and in bold.

Sensitivity analysis of by changing different models got similar results for all the outcomes in direct comparisons. Sensitivity analysis of direct comparisons by excluding each study one by one was consistent with the former results. Heterogeneity of direct comparisons was high in the rigorous BP vs. standard BP group, because the included studies used log transformations in the results. Heterogeneity in the network comparisons was mainly from the ACEI-ARB-CCB loop, so we checked the heterogeneity through the node-splitting (Table 10). There was no significant heterogeneity in the node-splitting.

**Table 10:** The heterogeneity of the antihypertensive treatments in the network comparisons

Name	Direct Effect	Indirect Effect	Overall	*P*-Value
Node-splittings of eGFR				
ACEI, ARB	−3.68 (−31.24, 24.03)	18.73 (−16.14, 54.44)	5.88 (−18.72, 26.54)	0.27
ACEI, CCB	13.24 (−12.01, 38.58)	−10.33 (−46.59, 27.04)	6.23 (−15.48, 26.17)	0.27
ARB, CCB	−6.05 (−30.78, 19.10)	15.85 (−20.44, 52.61)	0.22 (−20.78, 22.72)	0.30
Node-splittings of Scr				
ACEI, ARB	−0.07 (−0.56, 0.44)	−0.46 (−1.40, 0.46)	−0.16 (−0.57, 0.25)	0.40
ACEI, CCB	−0.01 (−0.54, 0.51)	0.38 (−0.52, 1.28)	0.04 (−0.35, 0.53)	0.41
ARB, CCB	0.44 (−0.28, 1.19)	0.06 (−0.67, 0.79)	0.21 (−0.25, 0.72)	0.41
Node-splittings of UAE				
ACEI, ARB	−22.14 (−198.76, 152.03)	−116.21 (−382.10, 163.42)	−29.95 (−145.72, 61.87)	0.42
ACEI, CCB	134.31 (−27.00, 289.65)	216.96 (−34.52, 464.40)	146.03 (47.16, 263.33)	0.47
ARB, CCB	234.56 (26.43, 437.04)	158.81 (−79.39, 396.13)	177.55 (74.83, 317.05)	0.49
Node-splittings of SBP				
ACEI, ARB	−1.85 (−6.79, 3.06)	3.78 (−12.61, 20.69)	−1.50 (−6.22, 3.18)	0.53
ACEI, CCB	4.90 (−1.19, 11.23)	0.42 (−16.72, 16.41)	4.70 (−1.30, 9.96)	0.58
ARB, CCB	2.58 (−12.98, 18.44)	6.87 (−1.02, 14.93)	6.17 (−1.00, 12.87)	0.65
Node-splittings of DBP				
ACEI, ARB	−0.91 (−6.63, 5.25)	4.05 (−17.77, 25.81)	−0.69 (−5.80, 4.95)	0.66
ACEI, CCB	3.05 (−4.94, 10.92)	−1.39 (−23.74, 18.97)	2.81 (−4.74, 9.45)	0.67
ARB, CCB	−0.15 (−22.20, 20.92)	3.78 (−6.36, 13.51)	3.44 (−5.65, 11.26)	0.73

## DISCUSSION

This study provided evidences for the antihypertensive treatments from 10 RCTs evaluating six interventions in adult patients with ADPKD. Overall, network comparisons and direct comparisons both indicated there was currently insufficient evidence of an association between lowering BP and the surrogate measures of kidney.

All the treatments did not differ in eGFR, Scr, SBP, DBP, MAP, and LVMI in network comparisons. Compared with β-blocker or CCB, RASIs did not show different effects on the renal function. ACEI was not associated with significantly protective effects on eGFR and UAE when compared with placebo. However, ACEI signiﬁcantly decreased SBP, DBP, MAP and LVMI when compared with CCB. Significantly increased UAE was observed in CCB compared with RASI treatments. No significant outcome was found in Dilazep compared with placebo. The rigorous BP control was associated with lower LVMI than the standard BP control. ARB may be relatively the most suitable treatment for eGFR, UAE and SBP in ADPKD.

RASIs are the first-line treatments for hypertension in ADPKD till now [16]. However, little beneﬁcial effect of RASIs in renal function was found in ADPKD patients in the past [17]. Therapeutic effects of RASI in renal function might be limited due to massive cystic involvement. EGFR in the majority of ADPKD patients remained steady until the late stage of the disease [18]. Combination of ACEI and ARB which was supposed to solve the compensatory feedback showed similar treatment effects of eGFR and TKV when compared with the ACEI monotherapy [1, 2].

UAE reflects the level of glomerular proteinuria, which is considered as a marker of glomerular injury [19]. ACEI is widely used in CKD to reduce the albuminuria mainly through lowering the intra-glomerular pressure [20]. Protective effects of ACEI were almost found in patients with chronic glomerulonephritis or proteinuria > 2 g/24h which did not always happen in ADPKD [21]. ADPKD patients were always accompanied with low levels of UAE (<2 g/24h). Therefore the anti-albuminuria effect of the ACEI still need large-scale studies to prove.

CCB was associated with increased UAE than RASIs [3, 8, 10]. We noticed that the CCB used in the trials was amlodipine (L-type CCB). CCBs varied in their effects of glomerular arterioles. T- or N-channel receptors mainly existed on the afferent and efferent arteriole, while L-channel receptors predominantly existed on the afferent arteriole. T-/N-channel blockade led to a reduction of intra-glomerular pressure and accordingly decreased UAE levels, while blockade of L-channel receptors led to an increase of UAE [22]. On the other hand, cytosolic Ca^2+^ depletion caused by *PKD1/2* mutation could activate cyclic adenosine monophosphate (cAMP) signal pathway and accelerate cystic proliferation in ADPKD [23, 24]. CCB might aggravate the Ca^2+^ depletion of the tubules and activate the cAMP pathway. However, this hypothesis needed to be testified.

Β-blockers treatment was limited and uncertain according to the existing outcomes. Β-blockers could inhibit RAAS activation by suppressing renin release. Evidence about β-blockers in ADPKD still needs more studies to prove.

LVMI is known as cardiovascular risk factor for morbidity or mortality in ADPKD patients [19]. Left ventricular hypertrophy frequently occurs in ADPKD patients with hypertension. LVMI decrease of hypertensive patients could bring benefits in reduced cardiovascular risk and mortality. Only rigorous BP control was found to be associated with obvious decline in LVMI compared with the standard BP control. Moreover, the HALT-PKD study found rigorous BP control could slow TKV significantly in the patients with early ADPKD [1, 2]. However, the eGFR and the UAE were not significant in the rigorous BP control group.

There were few data on patient relevant endpoints, such as end stage renal disease, need for dialysis/transplantation and mortality in addition to adverse drug effects. Zeltner et al. [4] reported no difference between ACEI vs. β-blocker in the need for dialysis/transplantation and the risk of cardiovascular events. Nutahara et al. [3] reported no difference between ARB vs. CCB in the risk of doubling of Scr.

This study had several limitations. First, the sample size of included studies was scant. Therefore, conclusions of eGFR and secondary outcomes were uncertain. Secondly, most of the ADPKD patients were prescribed with combination antihypertensive drugs. Our results were influenced inevitably by mixed drug effects. Thirdly, safety endpoints were poorly deﬁned in included studies. Moreover, this study could not assess subgroup analysis by different ADPKD genotypes (*PKD1&PKD2*) with different speed of renal progression.

In conclusion, this network meta-analysis is underpowered to detect differences of antihypertensive treatments on kidney progression in ADPKD patients. More RCTs and research about T-/N- type CCBs will be needed in the future. Use of ARB in clinical practice may be an optimal choice.

## MATERIALS AND METHODS

### Search strategy and selection criteria

We (X.C. and D.B.) searched PubMed, Embase, Ovid, and Cochrane Collaboration (published up to May, 2015) with the following terms: “angiotensin converting enzyme inhibitors”, “ACEIs”, “ACE inhibitors”, “angiotensin receptor blockers”, “ARBs”, angiotensin receptor antagonists”, “beta-blockers”, “β-blockers”, “beta-receptor antagonist”, “beta adrenergic antagonists”, “calcium antagonists”, “CCBs”, “calcium channel blockers”, “diuretics”, and the names of specific medications. The references of relevant reviews and clinical studies were checked in case of missed articles. We also searched the Google Scholar and clinical trials website.

### Inclusion and Exclusion Criteria

Included studies had to meet the following criteria: (1) studies in patients with the diagnosis of ADPKD; (2) antihypertensive drugs were used; (3) RCTs; (4) adults. Studies with the following criteria were excluded: (1) ADPKD patients with end stage renal disease or dialysis; (2) cohort studies or case-control studies.

### Data Extraction and methodological quality assessment

Two authors (X.C. and Z.C.) independently checked the included studies to extract the relevant data and assess study bias/risk. Discrepancy was settled by discussion. We evaluated the bias/risk of the included trials by using the Cochrane Risk of Bias Scale [25]. The primary outcome was estimated glomerular filtration rate (eGFR, mL/min or mL/min/1.73 m^2^). Secondary outcomes were serum creatinine (Scr, mg/dL), urinary albumin excretion (UAE, mg/d or mg/g), systolic blood pressure (SBP, mm Hg), diastolic blood pressure (DBP, mm Hg), mean artery pressure (MAP, mm Hg), left ventricular mass index (LVMI, g/m^2^).

### Statistical analysis

The meta-analysis was carried out according with the PRISMA extension statement for reporting of systematic review and network meta-analysis [26]. Heterogeneity was measured through Q test and *I^2^* statistics [27]. *I²* < 25% was considered as low and *I²* > 75% as high. We estimated the mean difference (MD) with 95% conﬁdence interval (CI) for the continuous calculations in the random effects model. Sensitivity analysis was estimated by the influence analysis which excluded each study to check the stability.

Network meta-analysis was performed by a Bayesian Markov Chain Morte Carlo method. Network meta-analysis needs to assume transitivity which holds when all direct comparisons between drugs have similar distribution of effect modiﬁers. The effect modiﬁers in this study included the BP at baseline, the level of eGFR, UAE, Scr and LVMI. All indirect treatment comparisons were taken together to get an integrated estimate of the included treatments. Different outcomes between direct and indirect evidences suggested that the assumption of transitivity might not depend. Included trials were grouped into six comparison categories: ACEI, ARB, ACEI+ARB, β-blocker, dilazep and CCB. Evaluation of inconsistency used the node-splitting. Network meta-analysis was calculated in both consistency and inconsistency models. Ranking of the drugs in each outcome was measured by Bayesian probability analysis. Software used were WinBUGS version 1.4 (Imperial College and Medical Research Council, London), Revman 5.4 (Cochrane group) and Stata version 13.1 (Stata Corp., College Station, Texas) [28].

## SUPPLEMENTARY MATERIAL FIGURES


