# Supplementary material for: Antihypertensive treatments in adult autosomal dominant polycystic kidney disease: network meta-analysis of the randomized controlled trials
Source: Oncotarget. 2015 Dec 2;6(40):42515–29. doi: 10.18632/oncotarget.6452 (PMC4767449; doi:10.18632/oncotarget.6452)
Supplement: Supplementary file 1 [file oncotarget-06-42515-s001.pdf]

Antihypertensive treatments in adult autosomal dominant polycystic kidney disease: network meta-analysis of the randomized controlled trials

Supplementary Material

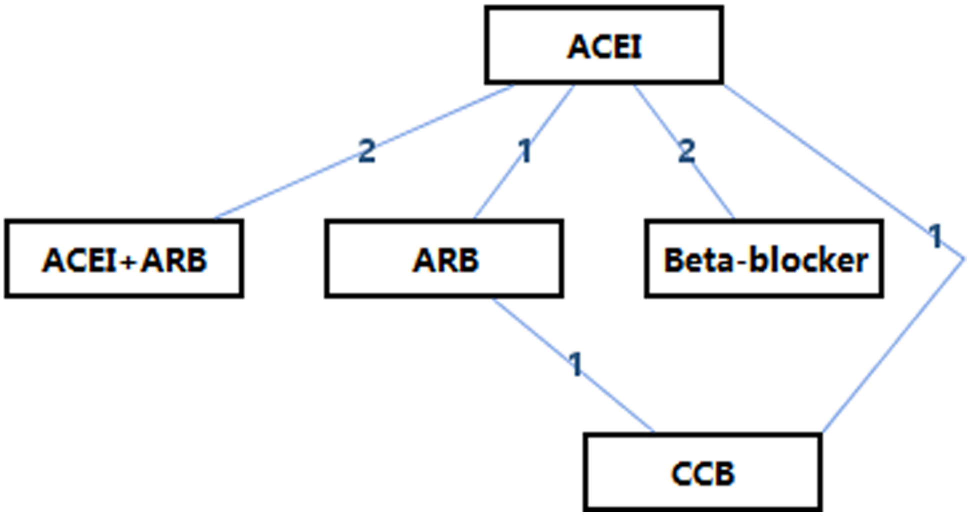

Supplemental Figure 1 Network of treatment comparisons for eGFR.

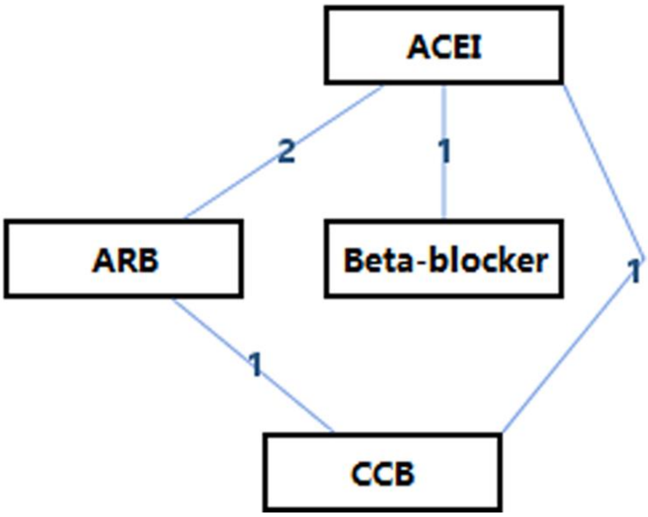

Supplemental Figure 2 Network of treatment comparisons for Scr.

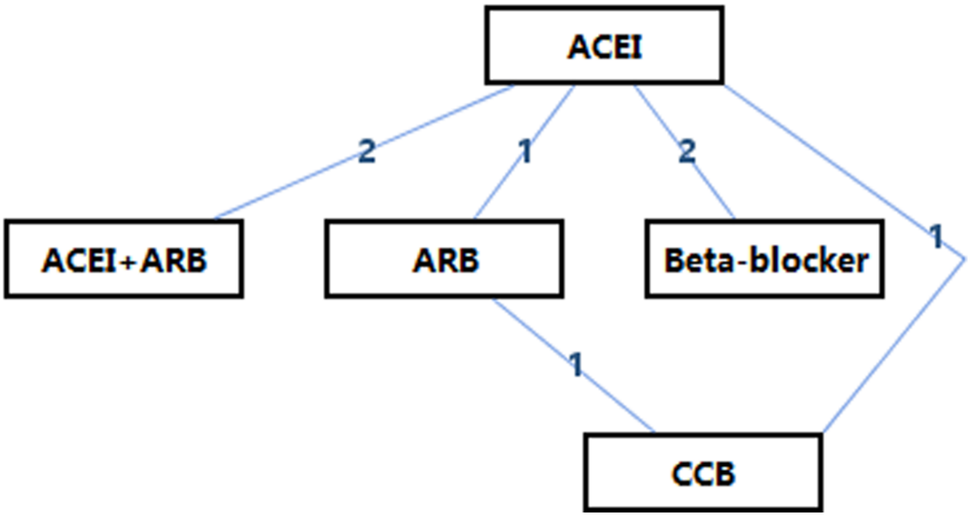

Supplemental Figure 3 Network of treatment comparisons for UAE.

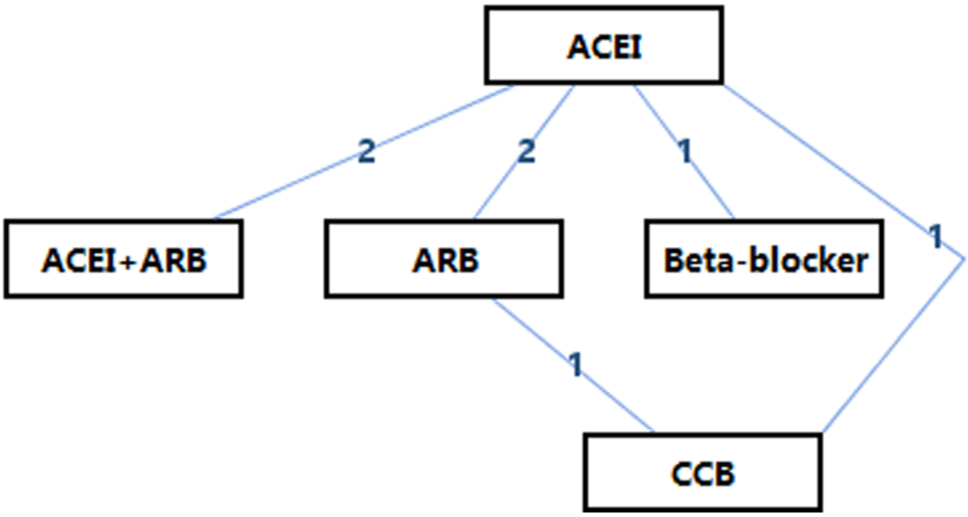

Supplemental Figure 4 Network of treatment comparisons for SBP.

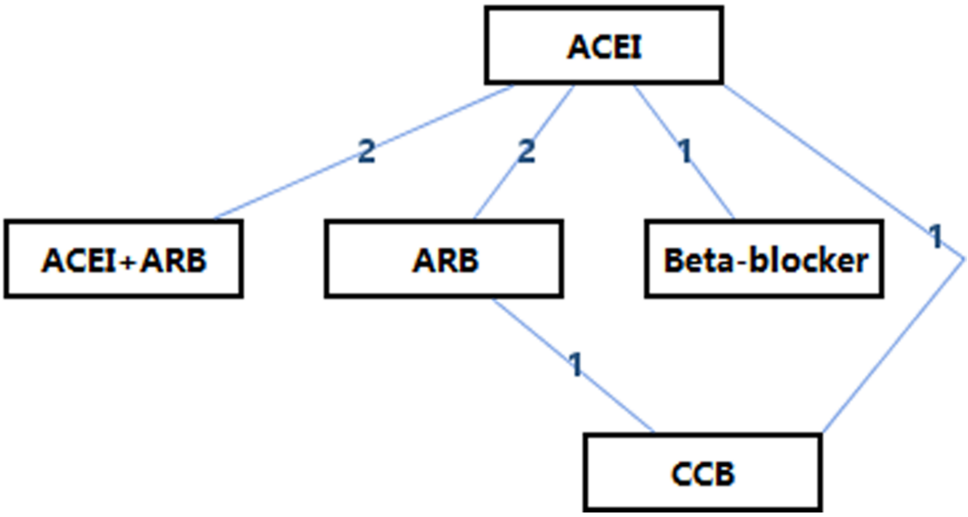

Supplemental Figure 5 Network of treatment comparisons for DBP.

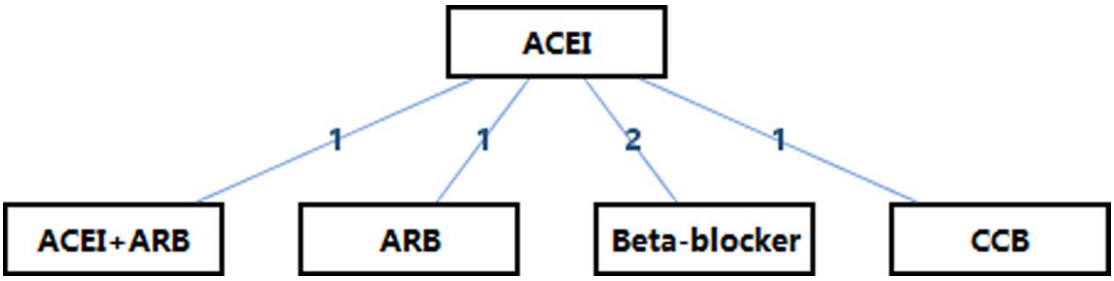

Supplemental Figure 6 Network of treatment comparisons for MAP.

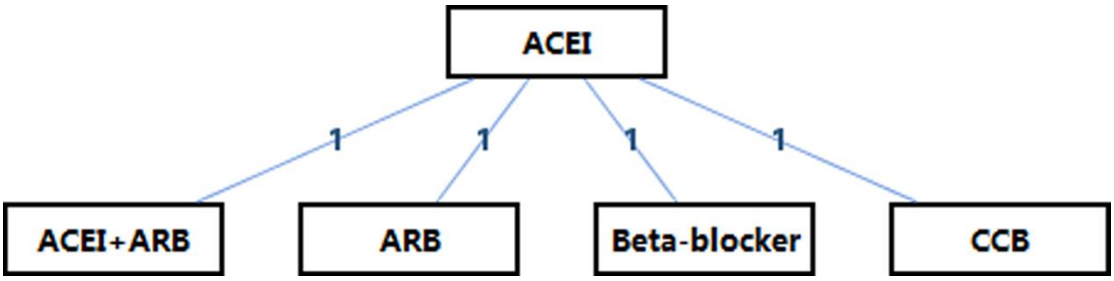

Supplemental Figure 7 Network of treatment comparisons for LVMI.
